# DNA Nanostructure-Assembled Metallic Nanoparticles for Biosensing Applications

**DOI:** 10.3390/molecules31030513

**Published:** 2026-02-02

**Authors:** Shaokang Ren, Kai He, Canlin Cui, Haoyu Fan, Hongzhen Peng, Kai Jiao, Lihua Wang

**Affiliations:** 1 Institute of Materiobiology, College of Sciences, Shanghai University, Shanghai 200444, China; iamskren@163.com (S.R.); hekai2511@163.com (K.H.); cuicanlin@shu.edu.cn (C.C.); nana7mi@shu.edu.cn (H.F.); penghongzhen@shu.edu.cn (H.P.); 2 Shanghai Collaborative Innovation Center of Intelligent Sensing Chip Technology, Shanghai University, Shanghai 200444, China

**Keywords:** DNA nanostructures, metal nanoparticles, plasmonic properties, biosensing

## Abstract

DNA nanotechnology offers an unprecedented level of structural programmability for organizing metallic nanoparticles into precisely defined architectures, providing a powerful platform for plasmonic biosensing. In particular, gold and silver nanoparticles assembled on DNA nanostructures enable nanometer-scale control over interparticle distance, orientation, and spatial symmetry, which directly govern collective plasmonic behaviors and optical signal transduction. This review summarizes recent advances in DNA nanostructure-mediated assembly of metal nanoparticles, with an emphasis on design principles and assembly strategies that enable static and dynamic control of nanoparticle organization. Representative examples are discussed to illustrate how well-defined plasmonic assemblies give rise to tunable optical responses, including localized surface plasmon resonance modulation, chiroptical signals, fluorescence enhancement or quenching, and surface-enhanced Raman scattering. The role of structural programmability and stimulus-responsive reconfiguration in translating molecular recognition events into amplified optical outputs is highlighted in the context of biosensing. Finally, current challenges and future perspectives are outlined, focusing on structural robustness, signal reproducibility, and integration toward practical and multiplexed biosensing platforms.

## 1. Introduction

The development of highly sensitive, specific, and multiplexed biosensors has become increasingly important for biomedical diagnostics, environmental monitoring, and point-of-care applications. Achieving high sensor performance requires not only accurate molecular recognition but also precise control over signal transduction processes [1,2]. Metallic nanoparticles, particularly gold and silver nanoparticles, have emerged as key functional nanomaterials due to their unique plasmonic properties. Their size- and shape-dependent optical responses enable enhanced sensitivity, tunable signal outputs, and versatile detection modalities, making them widely employed in biosensing platforms [3,4].

To fully exploit these properties, metallic nanoparticles must be organized into well-defined architectures. The spatial positions, orientations, and interparticle distances critically influence collective plasmonic effects, electromagnetic field enhancement, and electron transfer pathways, all of which directly impact signal amplification and detection sensitivity [5,6,7]. Well-controlled assemblies therefore offer improved reproducibility, enhanced signal output, and opportunities for integrating multiple sensing functions, which are difficult to achieve with randomly distributed nanoparticles.

However, conventional nanoparticle assembly strategies often suffer from limited spatial precision, structural heterogeneity, and poor batch-to-batch consistency [8,9,10]. Random aggregation or nonspecific adsorption can lead to heterogeneous structures, uncontrolled interparticle distances, and variable signal outputs, which significantly hinder sensor performance. Overcoming these challenges requires scaffolds capable of directing nanoparticle assembly with nanometer-scale accuracy while offering programmability and dynamic responsiveness.

DNA nanotechnology provides a versatile solution to these challenges [11,12]. Through the rational design of DNA tiles [13,14,15,16,17], origami [18,19,20,21,22], and wireframe structures [23,24], nanoparticles can be positioned at defined locations with sub-10 nm precision. Importantly, DNA nanostructures function not only as passive templates but also as active components that regulate nanoparticle spatial organization, plasmonic coupling, and signal transduction, functioning as integral components of functional biosensors. This approach enables programmable, reproducible, and multifunctional assemblies that can be dynamically reconfigured in response to specific molecular inputs.

While numerous excellent reviews have summarized biosensors based on DNA-functionalized metallic nanoparticles [25,26], this review focuses specifically on gold and silver nanoparticles assembled through DNA nanostructures, emphasizing design principles and assembly strategies, with representative examples illustrating signal transduction mechanisms and biosensing applications. We also discuss current challenges and future directions for integrating DNA nanostructures with gold and silver nanoparticles to construct high-performance, programmable biosensors.

## 2. DNA Nanostructures as Programmable Scaffolds

**Figure 1:**
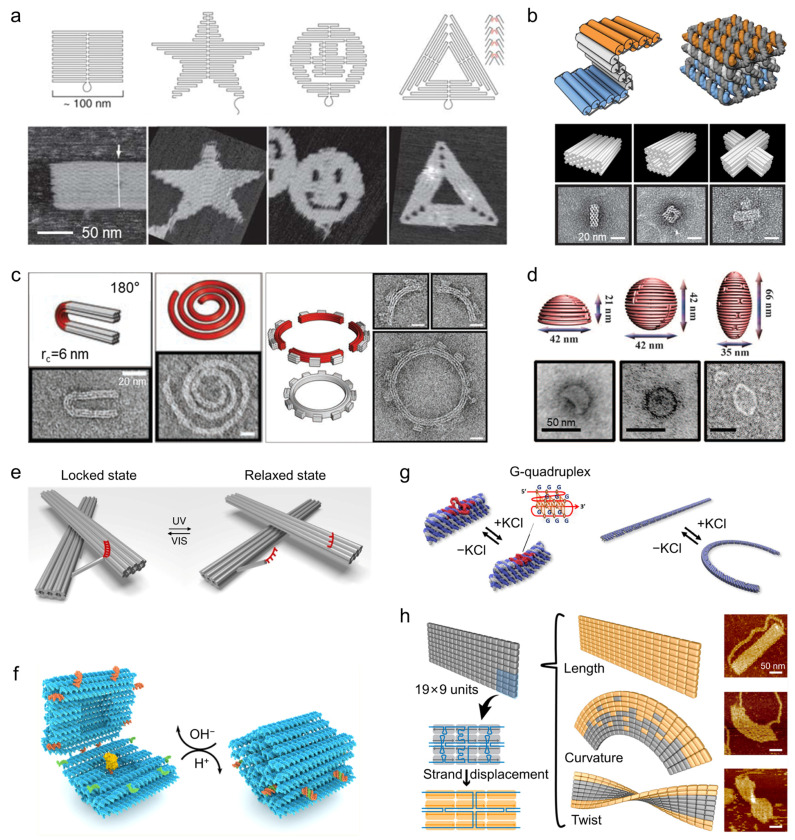
DNA origami nanostructures. (**a**) Symmetric 2D monolayer DNA origami structures: rectangle, five-pointed star, smiley face, and triangle (left to right) [31]. Copyright 2024, American Chemical Society. (**b**) 3D multilayer DNA origami structures with honeycomb packing [32]. Copyright 2016, Wiley. (**c**) 3D DNA origami structures exhibiting complex curvature [20]. Copyright 2009, AAAS. (**d**) DNA nanostructures with gradually varying radii, including hemispheres, spheres, and ellipsoids [22]. Copyright 2011, AAAS. (**e**) Conformational transitions of DNA origami mediated by light-responsive components [33]. Copyright 2016, Springer Nature. (**f**) Opening and closing of DNA nanocapsules regulated by pH-responsive modules. Green and orange represent the pH response module, while yellow represents the cargo inside the nanostructure [34]. Copyright 2019, American Chemical Society. (**g**) Global deformation of DNA origami via metal ion-responsive G-quadruplex motifs [35]. Copyright 2020, Wiley. (**h**) Tuning of length, curvature, and twist in DNA origami through toehold-mediated strand displacement [36]. Copyright 2021, American Chemical Society.

DNA nanotechnology provides an unprecedented platform for constructing highly precise and programmable nanoscale architectures, enabling the controlled organization of metallic nanoparticles [27,28]. DNA origami, in particular, has become a cornerstone technique in this field, offering a versatile and robust scaffold for the assembly of complex nanostructures. Initially introduced by Rothemund in 2006, the scaffolded DNA origami technique uses a long single-stranded DNA scaffold, which is folded into well-defined two-dimensional (2D) shapes through hybridization with hundreds of short staple strands in a one-pot annealing reaction [18]. This method enables the fabrication of planar structures up to ~100 nm in size with ~6 nm resolution, facilitating the creation of a wide variety of shapes, such as rectangles, stars, smiley faces, and triangles (Figure 1a). The simplicity, low cost, and modularity of this approach quickly spurred more complex designs, including asymmetric forms like a map of China [29], curved concentric rings [22], and even a Möbius strip [30].

Building on this 2D foundation, DNA nanostructures have been extended into three dimensions through techniques such as multi-layer stacking of DNA helices. Notable examples include the work by Douglas et al., who created a range of three-dimensional (3D) objects such as monoliths, square nuts, railed bridges, genie bottles, stacked crosses, and slotted crosses using a honeycomb-pleated lattice, with tunable dimensions between 10–100 nm (Figure 1b) [21]. Subsequently, Dietz et al. advanced the design by introducing precise, programmable bending and twisting of 3D nanostructures via base-pair insertions and deletions, resulting in intricate shapes like wireframe beach balls, gears, and spirals (Figure 1c) [20]. Additionally, Han et al. demonstrated a curvature-centric approach to designing 3D structures, coupling longitudinal and latitudinal bending to assemble smoothly curved geometries such as hemispheres, spheres, and nanoflasks, thus expanding the structural repertoire beyond angular, raster-based designs (Figure 1d) [22].

A significant leap in DNA nanotechnology has been the integration of dynamic and stimuli-responsive elements into otherwise static structures, transforming them into adaptive, responsive systems. For instance, Kuzyk et al. developed a light-driven reconfigurable DNA nanostructure by incorporating azobenzene-modified strands [33]. The photo-induced switching between trans and cis states triggered a macroscopic conformational change, which was amplified ~100-fold from the molecular length change (Figure 1e). Additionally, pH-responsive DNA structures have been realized by Ijäs et al., who designed a nanocapsule that opens at high pH for cargo loading and closes via Hoogsteen triplex formation at low pH, encapsulating gold nanoparticles in the process (Figure 1f) [34]. Ion-triggered reconfiguration was demonstrated by Suzuki, who constructed a nanoarm based on G-quadruplex-containing modules. This nanoarm contracts and relaxes reversibly upon the addition of K^+^, toggling between linear and arched states (Figure 1g) [35].

Further enhancing the versatility of DNA scaffolds, modular reconfiguration has also been explored. Wang et al. introduced a modular expandable origami system wherein selective strand-displacement reactions allow for the independent adjustment of local length, curvature, and twist of a 2D sheet, enabling the controlled morphing of the shape (Figure 1h) [36]. This strategy demonstrates the potential for building more sophisticated, programmable DNA nanostructures that can dynamically adapt to specific environmental cues or functional requirements.

These advances in static and dynamic DNA nanostructures illustrate the profound potential of DNA nanotechnology as a programmable scaffold for organizing nanoparticles with high precision. By leveraging these DNA scaffolds, researchers can create nanoparticle assemblies that are not only spatially defined but also able to respond to external stimuli, enabling new opportunities in biosensing, where dynamic control over the structure and behavior of nanoparticle assemblies is crucial.

## 3. DNA Nanostructure-Mediated Assembly of Metal Nanoparticles

**Figure 2:**
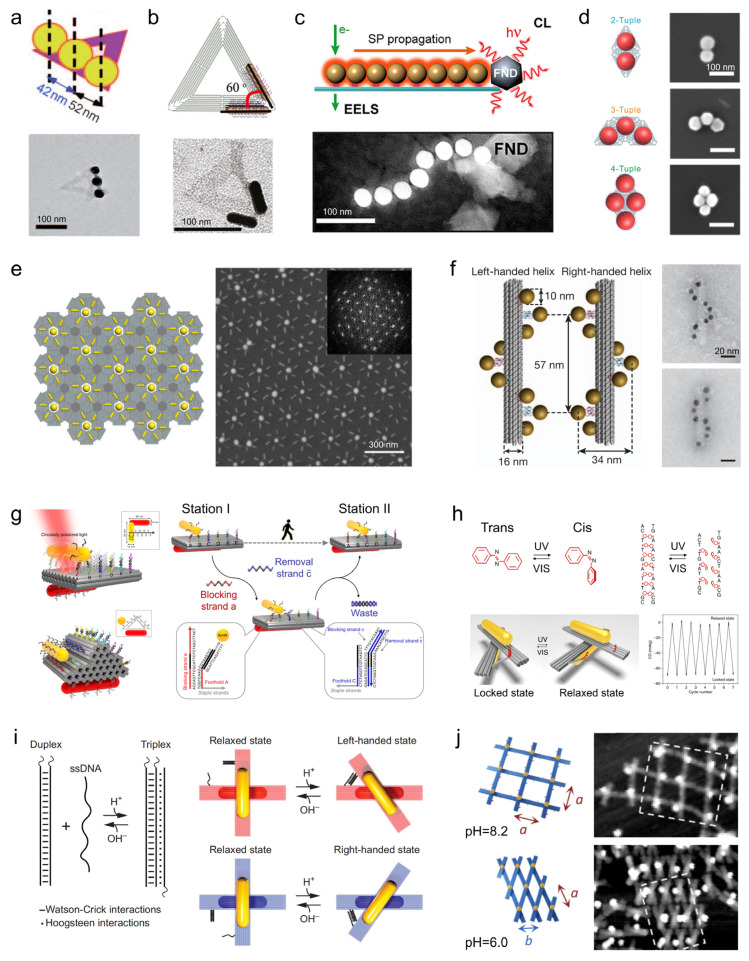
DNA Nanostructure-Mediated MNP architectures. (**a**) Precise control over the position, number, and spacing of AgNPs using triangular DNA origami [38]. Copyright 2010, Wiley. (**b**) Angle regulation of AuNRs based on triangular DNA origami templates [39]. Copyright 2011, American Chemical Society. (**c**) DNA-origami-based self-assembly of gold nanoparticle chains for plasmonic waveguides [40]. Copyright 2018, American Chemical Society. (**d**) AuNP metamolecules based on super-origami templates [41]. Copyright 2019, AAAS. (**e**) Fabrication of chiral metasurface via microscale DNA origami array [42]. Copyright 2025, American Chemical Society. (**f**) Construction of helical AuNP assemblies with optical chirality responses utilizing DNA origami 24-helix bundles [44]. Copyright 2018, American Chemical Society. (**g**) A DNA origami-based AuNR walker system driven by toehold-mediated DNA strand displacement reactions [45]. Copyright 2015, Springer Nature. (**h**) A light-responsive DNA origami template to construct a plasmonic chiral nanosystem [33]. Copyright 2016, Springer Nature. (**i**) Reconfigurable chiral plasmonic metamolecules fabricated using pH-sensitive dynamic DNA origami [46]. Copyright 2017, AAAS. (**j**) Dynamic modulation of AuNP lattice configurations through reconfigurable pH-responsive DNA origami lattices [47]. Copyright 2023, American Chemical Society.

Building on the structural programmability of DNA nanostructures, gold and silver nanoparticles can be assembled into well-defined architectures with precise spatial control. At the most fundamental level, individual geometric parameters, such as interparticle distance and angular orientation [37], can be tuned to modulate plasmonic interactions. For example, silver nanoparticle dimers assembled on triangular DNA origami achieved center-to-center distances of ~90 nm, ~49 nm, and ~24 nm by adjusting the positions of capture strands (Figure 2a), illustrating nanometer-scale control over near-field coupling [38]. Angular orientation can also be precisely regulated, as shown by gold nanorod dimers on DNA origami templates, where 0°, 60°, 90°, and 180° configurations with gaps down to ~5 nm were realized (Figure 2b), enabling directional plasmonic responses [39].

Extending this precise control to multi-particle assemblies allows the creation of hierarchical arrays. One-dimensional arrangements of nanoparticles with uniform spacing form plasmonic waveguides capable of directing energy to distal reporters, exemplified by DNA-guided nanowire systems (Figure 2c) [40]. Two-dimensional lattices constructed with controlled interparticle spacing generate collective interference effects, such as Fano resonances, enhancing surface-enhanced Raman scattering (SERS) sensitivity (Figure 2d) [41]. Further, by positioning multiple DNA origami units in defined orientations on a surface, these patterned assemblies can form superlattices or metasurfaces, providing a structural basis for exploring nonlinear optical responses and collective field enhancement in macroscopic arrays (Figure 2e) [42].

Beyond planar configurations, three-dimensional assembly strategies expand the functional repertoire. By arranging nanoparticles into left- or right-handed helices, DNA scaffolds produce pronounced chiroptical responses, including circular dichroism (CD) signals (Figure 2f) [43]. Such 3D architectures not only increase structural diversity but also directly contribute to biosensing, as molecular recognition events can induce measurable changes in the chiral configuration.

Collectively, these hierarchical DNA-mediated assembly strategies demonstrate the versatility of DNA nanostructures in guiding nanoparticles from precise control of interparticle distance and angle, through one- and two-dimensional patterning, to three-dimensional helical architectures. This programmability enables the construction of plasmonic waveguides, Raman-enhancing lattices, and metasurfaces with potential for nonlinear optical applications, while simultaneously supporting sensitive, tunable, and multifunctional biosensing platforms.

While static DNA origami architectures provide precise and robust control over nanoparticle spacing and geometry, thereby maximizing plasmonic coupling and signal intensity, the incorporation of dynamic, stimuli-responsive elements enables these assemblies to actively transduce molecular inputs into measurable optical signals. In biosensing contexts, such reconfigurability is not merely structural but functional, as it directly translates molecular recognition events into signal amplification, reversibility, and background suppression.

A representative mechanism for dynamic signal transduction is toehold-mediated strand displacement, which enables programmable and reversible repositioning of nanoparticles on DNA scaffolds. Zhou et al. demonstrated an active plasmonic “walker” system in which a gold nanorod moved stepwise along a DNA track with ~7 nm spacing via strand displacement (Figure 2g) [45]. Importantly, the walker motion induced continuous and reversible changes in circular dichroism, illustrating how molecular inputs can be converted into real-time plasmonic signal outputs. Such systems exemplify how dynamic reconfiguration enables signal switching and temporal control, rather than static signal readout.

Light-responsive DNA origami further highlights how external stimuli can modulate plasmonic responses with high spatiotemporal precision. Kuzyk et al. developed a reconfigurable DNA origami template in which azobenzene-modified hinges allowed reversible switching between locked and relaxed states under UV/Vis illumination [33]. This molecular-scale actuation was amplified into a ~30 nm structural displacement, resulting in pronounced changes in chiral plasmonic responses in gold nanorod assemblies (Figure 2h). The ability to reversibly toggle plasmonic signals without chemical reagents offers clear advantages for background suppression and repeated sensing cycles.

Chemical stimuli such as pH provide an additional route for dynamic plasmonic modulation. By replacing azobenzene hinges with pH-sensitive DNA locks, Kuzyk et al. achieved reversible switching of plasmonic chirality in DNA-based metamolecules (Figure 2i) [46]. Similarly, Julin et al. designed pH-responsive DNA origami units whose opening and closing triggered large-scale reorganization of nanoparticle arrays, altering interparticle distances and lattice geometry (Figure 2j) [47]. In both cases, environmental changes were directly translated into distinct plasmonic states, enabling signal gating and condition-dependent readout.

Collectively, these examples illustrate how static DNA origami structures function as plasmonic signal amplifiers by defining optimal nanoparticle arrangements, whereas dynamic DNA nanotechnology enables signal transduction by converting molecular or environmental stimuli into reversible optical responses. The integration of static amplification with dynamic reconfiguration thus provides a powerful framework for biosensing, allowing high sensitivity, low background, and real-time adaptability within a single DNA-programmed plasmonic platform.

## 4. Tunable Plasmonic Properties of DNA Nanostructure-Mediated Metal Nanoparticle Assemblies

The integration of metallic nanoparticles with DNA nanostructures has established a powerful paradigm for plasmonic material design, in which the structural programmability of DNA scaffolds is directly translated into tunable optical responses. By enabling nanometer-scale control over interparticle distance, spatial orientation, symmetry, and local dielectric environment, DNA nanostructures provide a unique platform for engineering plasmonic coupling phenomena that are difficult to access using conventional top-down fabrication strategies. This precise control over nanoscale geometry allows collective plasmonic modes to be deliberately designed and optimized, giving rise to well-defined optical outputs.

As a result, key plasmonic signal modalities relevant to biosensing, including CD, fluorescence modulation, and SERS can be systematically regulated through rational structural design. Importantly, DNA nanostructures can function either as static architectural frameworks that maximize electromagnetic field enhancement or, when combined with reconfigurable motifs, as dynamic platforms capable of converting molecular inputs into optical signals. Together, these DNA nanostructure-based plasmonic systems enable biosensing architectures that combine strong signal amplification with programmable optical signal transduction [48,49,50].

### 4.1. Chiroptical Modulation

Chiroptical responses are among the most geometry-sensitive plasmonic phenomena, making them particularly suitable for elucidating how DNA nanostructures mediate plasmonic mode engineering. DNA nanostructures enable the precise construction of chiral plasmonic assemblies by offering nanometer-level control over nanoparticle positioning, relative orientation, and global symmetry. This structural precision allows the realization of a broad range of geometrically chiral architectures, including helices, asymmetrically positioned dimers, and cross-stacked oligomers, which collectively give rise to pronounced plasmonic CD signals in the visible and near-infrared regions.

**Figure 3:**
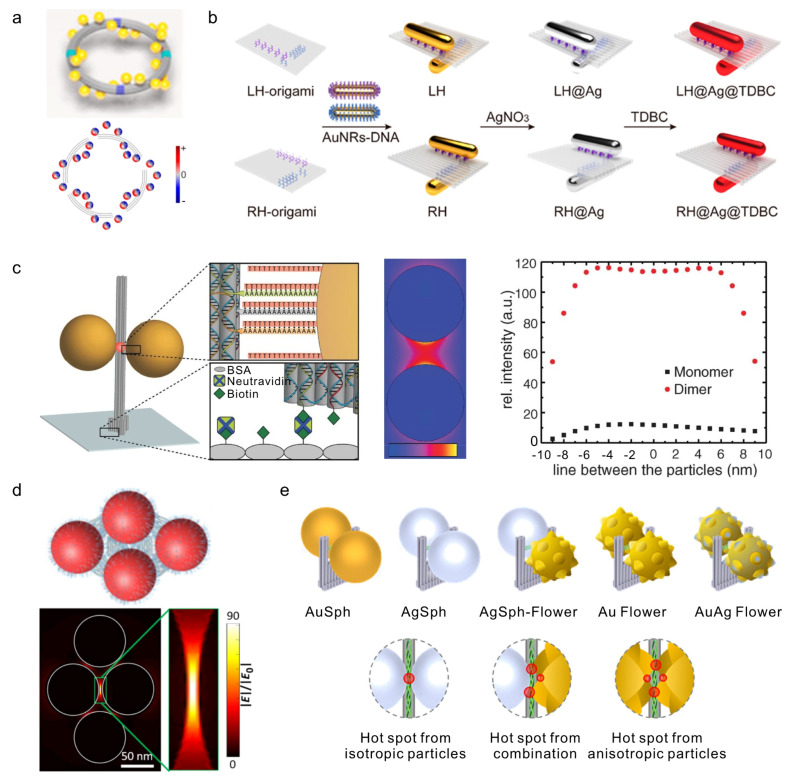
Tunable optical properties of DNA plasmonic nanostructure assemblies. (**a**) DNA origami-templated gold nanoparticle helices exhibiting strong CD due to geometry-controlled collective plasmonic coupling [51]. Copyright 2016, American Chemical Society. (**b**) Chiral plasmonic–excitonic hybrids, where gold nanorod dimers on DNA scaffolds interact with excitonic dyes to produce enhanced, tunable CD responses. The purple and blue lines represent the assembly sites of gold nanorod [53]. Copyright 2021, American Chemical Society. (**c**) DNA origami nanoantennas positioning fluorophores in nanoparticle dimers, generating strong and reproducible fluorescence enhancement at electromagnetic hotspots [56]. Copyright 2012, AAAS. (**d**) Tetrameric gold nanoparticle assemblies on DNA origami forming Fano-resonant SERS hotspots, enabling quantitative Raman measurements with controlled reporter placement [41]. Copyright 2019, AAAS. (**e**) SERS optimization through DNA-directed control of nanoparticle composition and morphology, showing how geometric precision and material choice enhance local field confinement [57]. Copyright 2023, American Chemical Society.

A representative example is provided by the work of Urban and co-workers, who arranged gold nanoparticles into well-defined left- or right-handed helices on ring-shaped DNA origami templates to construct plasmonic toroidal metamolecules. Finite-element simulations faithfully reproduced the experimentally observed CD spectra and provided mechanistic insight into their physical origin. Analysis of the induced charge and current distributions revealed that, at plasmonic resonance, strong electromagnetic coupling between adjacent nanoparticles leads to the formation of collective plasmonic modes. The symmetry and handedness of these modes are dictated by the DNA-encoded chiral geometry, resulting in distinct interactions with left- and right-circularly polarized light and, consequently, pronounced CD responses (Figure 3a) [51]. These studies establish a clear structure–property relationship linking DNA nanostructures programmed geometry, collective plasmonic coupling, and emergent chiroptical activity.

Beyond purely geometric effects, DNA nanostructure-templated chiral assemblies also offer opportunities for further tuning chiroptical responses through material and hybridization strategies. For example, coating gold nanoparticles with silver shells enables precise adjustment of plasmonic resonance energies, while incorporation of excitonic components such as J-aggregate dyes introduce plasmon–exciton coupling (Figure 3b) [52,53]. In such hybrid systems, the interplay between chiral plasmonic modes and excitonic transitions leads to enhanced and spectrally tunable CD signals, highlighting how DNA nanostructures provide a modular framework for engineering complex chiroptical responses.

While many of these studies focus on static chiral architectures to maximize CD signal strength, the same design principles can be extended to reconfigurable DNA scaffolds, in which controlled structural rearrangements translate into switchable chiroptical outputs [54,55]. This capability forms the basis for using plasmonic CD as an optical readout in responsive sensing systems.

### 4.2. Fluorescence Enhancement and Energy Transfer Control

Fluorescence-based plasmonic readouts offer a complementary perspective by probing highly localized electromagnetic field enhancement at the single-emitter level. DNA nanostructures serve as precise scaffolds for positioning fluorophores within plasmonic nanocavities, enabling systematic regulation of excitation rates, emission intensity, polarization, and directionality through controlled light-matter interactions.

DNA origami-based plasmonic nanoantennas, typically composed of gold nanoparticle or nanorod dimers, have been extensively employed to investigate fluorescence enhancement mechanisms (Figure 3c). By placing fluorophores at predefined docking sites within nanogaps, local electromagnetic fields can be strongly enhanced, leading to fluorescence enhancement factors exceeding two orders of magnitude [56]. Such static architectures function as efficient optical amplifiers, providing reproducible and quantifiable enhancement of weak fluorescence signals.

Beyond excitation enhancement, plasmonic nanocavities also modify emission dynamics by reshaping the local density of optical states [58]. Single-molecule measurements reveal pronounced reductions in fluorescence lifetime, indicative of Purcell-enhanced radiative decay. Importantly, the balance between radiative enhancement and nonradiative energy dissipation is critically dependent on emitter–metal separation. The nanometer-scale distance control afforded by DNA origami enables fluorophores to be positioned within an optimal regime where radiative processes dominate over quenching, allowing predictable and rational design of fluorescence-modulating plasmonic structures.

Although most fluorescence enhancement studies employ static DNA scaffolds to maximize signal amplification, incorporation of stimulus-responsive DNA elements enables reversible modulation of emitter–nanoparticle coupling. In such cases, fluorescence intensity and lifetime can serve as optical reporters of DNA conformational states, providing a direct link between nanoscale structural changes and measurable optical outputs.

### 4.3. Surface-Enhanced Raman Scattering

Surface-enhanced Raman scattering represents an extreme manifestation of plasmonic field confinement, relying on highly localized electromagnetic hotspots to amplify intrinsically weak molecular vibrational signals. DNA origami provides an exceptional platform for SERS studies by enabling precise control over hotspot geometry, plasmonic mode structure, and molecular positioning [49,59,60].

As demonstrated by Fang and co-workers, gold nanoparticles can be programmably assembled into well-defined tetrameric superstructures using DNA origami super-frame designs (Figure 3d) [41]. These assemblies support Fano resonances arising from interference between super-radiant bright modes and sub-radiant dark modes. Electromagnetic simulations based on finite-difference time-domain and finite-element methods reveal that, at the Fano dip wavelength, radiative losses are suppressed and electromagnetic energy is strongly confined within interparticle gaps, resulting in extremely high local field enhancements. Crucially, the addressability of DNA origami allows a defined number of Raman reporter molecules to be positioned precisely within these computationally identified hotspots. Experimentally, SERS intensities exhibit stepwise, quantized increases with increasing molecular occupancy, directly linking signal strength to molecular number. This level of control transforms SERS from a stochastic enhancement phenomenon into a predictable and quantitative process governed by DNA-programmed plasmonic architecture.

Additional studies further demonstrate that nanoparticle composition and morphology play critical roles in determining SERS performance. Systematic comparisons of gold, silver, and anisotropic nanostructures assembled on DNA origami, supported by large-scale statistical analyses and electromagnetic simulations, show that plasmonic resonance positions and local field enhancements can be optimized through rational material selection and precise geometric control (Figure 3e) [57]. In this context, DNA nanostructures act as nanoscale photonic engineering platforms that enable reproducible and high-performance SERS substrates.

Taken together, these studies demonstrate that DNA nanostructures enable plasmonic properties to be engineered at a mechanistic level through precise control over nanoscale geometry and electromagnetic coupling. By enforcing well-defined interparticle spacing, orientation, and symmetry, DNA-guided assemblies improve the reproducibility of plasmonic architectures and, consequently, the consistency of their optical signal outputs. While different optical modalities exhibit varying sensitivity to nanoscale precision, a clear correlation between structural fidelity and signal reproducibility is generally observed. Collective chiroptical signals are relatively tolerant to minor geometric variations, whereas SERS readouts impose stricter requirements on hotspot definition.

Within this framework, static DNA-templated plasmonic architectures primarily function as efficient signal amplifiers by maximizing electromagnetic field enhancement, while the incorporation of dynamic DNA nanotechnology introduces responsiveness, allowing plasmonic systems to actively transduce molecular inputs into optical outputs. The integration of static amplification and dynamic signal transduction provides a versatile foundation for biosensing, enabling molecular recognition events to be both strongly amplified and reliably converted into quantitative optical readouts.

## 5. Biosensing Applications of DNA Nanostructure-Mediated Metal Nanoparticle Assemblies

The integration of DNA nanostructures with metal nanoparticles provides a versatile strategy for translating molecular recognition events into highly sensitive optical signals. Owing to their inherent programmability, DNA nanostructures enable the precise spatial organization of metal nanoparticles and optical emitters, yielding assemblies with finely tunable chiroptical, fluorescence, and Raman responses. Importantly, these optical signals can be dynamically modulated in response to specific biochemical interactions, allowing DNA nanostructure-based plasmonic systems to function as powerful transducers for molecular diagnostics and environmental biosensing [61,62].

### 5.1. Chirality-Based Biosensing

DNA nanostructure-templated chiral plasmonic assemblies are particularly effective at converting molecular recognition events into measurable variations in CD, providing a label-free and real-time sensing modality. By arranging AuNPs or AuNRs on DNA origami scaffolds with well-defined asymmetric, twisted, or crossed geometries, controllable chiroptical signals can be generated. Hybridization with complementary nucleic acid targets or binding of aptamer–ligand pairs induce conformational changes in the DNA framework, thereby modulating plasmonic coupling and producing distinct CD intensity or sign variations.

Using this principle, twisted AuNR dimers assembled through DNA hybridization have been shown to generate CD responses that correlate linearly with target DNA concentration, achieving detection limits as low as 3.7 aM [63]. Extending this concept to intracellular environments, side-by-side AuNR assemblies positioned within living cells enabled real-time monitoring of microRNA recognition via in situ CD signal changes, demonstrating the feasibility of chiroptical sensing in complex biological contexts [64].

Beyond static sensing, reconfigurable chiral plasmonic systems based on DNA origami have demonstrated broad applicability for detecting diverse targets, including nucleic acids, proteins, and small molecules [65,66,67]. Upon specific molecular recognition, these architectures undergo programmable conformational transitions that alter their chiral configuration, yielding quantifiable CD readouts. For example, cross-arranged AuNR dimers on DNA origami scaffolds exhibited reversible chirality switching upon viral RNA hybridization, enabling quantitative detection with limits of approximately 100 pM [65]. Similarly, aptamer-integrated chiral origami assemblies have been engineered to recognize ligands such as adenosine and thrombin, where target binding induces structural reorganization of the plasmonic framework, with dissociation constants down to 1.4 nM [67].

**Figure 4:**
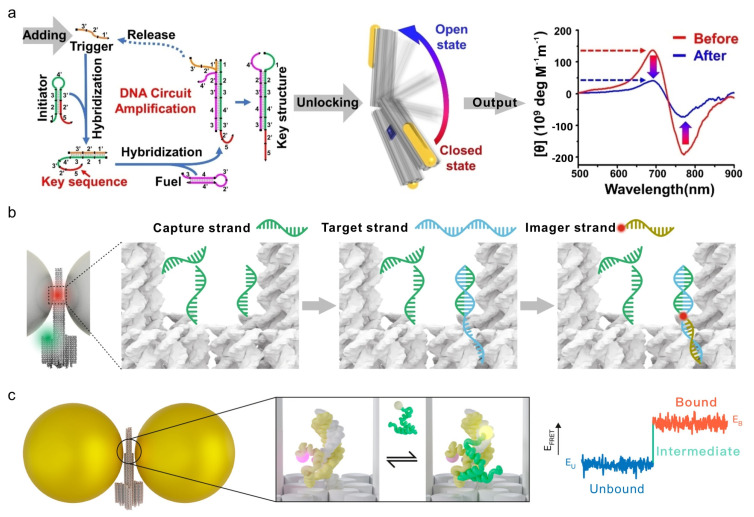
Chirality- and fluorescence-based biosensing. (**a**) A DNA amplification circuit converts the target analyte into a key structure, triggering a conformational transformation of the plasmonic assembly and generating a corresponding CD signal. The red line segment represents the key sequence that interacts with the nanotweezers [68]. Copyright 2022, Wiley. (**b**) Capture strands immobilized on a nanoantenna recognize and bind target molecules, exposing a hybridization site for an imaging strand that produces a radiometric fluorescence signal [69]. Copyright 2021, Springer Nature. (**c**) DNA origami-based nanoantennas enable single-molecule investigations of the coupled folding and binding dynamics of proteins [70]. Copyright 2024, American Chemical Society.

To further enhance sensitivity toward low-abundance biomarkers, signal amplification modules have been integrated upstream of the chiroptical readout. In representative designs, DNA amplification circuits recycle trace amounts of target molecules to generate abundant trigger strands, which subsequently induce large-scale reconfiguration of AuNR-functionalized DNA origami nanotweezers. This strategy effectively amplifies weak molecular recognition events into macroscopic CD responses, enabling detection of tumor-related mRNAs, adenosine, and cell-surface proteins at picomolar concentrations or even at the single-cell level (Figure 4a) [68]. Together, these examples highlight the potential of DNA nanostructure-based chiral plasmonic systems for ultrasensitive, real-time bioanalysis.

### 5.2. Fluorescence-Based Biosensing

The nanometer-scale spatial control afforded by DNA nanostructures provides an ideal platform for plasmon-enhanced fluorescence biosensing, where the radiative properties of fluorophores can be precisely modulated. DNA templates enable the construction of optical nanoantennas that translate molecular recognition events into amplified fluorescence signals.

Seminal work by Tinnefeld and co-workers established DNA origami-based optical nanoantennas as robust systems for plasmon-enhanced fluorescence sensing. By systematically optimizing nanoparticle size, shape, material composition, and fluorophore positioning, these platforms enabled tunable fluorescence enhancement and single-molecule nucleic acid detection [50,69,71]. In a representative design, single 80 nm Ag nanoparticles were anchored on DNA origami templates in a molecular beacon-like configuration, resulting in fluorescence enhancement of several orders of magnitude upon target hybridization. Using this approach, Zika virus DNA and RNA sequences were sensitively detected directly in serum samples [71].

Further refinement led to the development of trident-shaped NanoAntennas with Cleared Hotspots (NACHOS), in which two Ag nanopillars defined a nanoscale junction containing capture strands. Upon target hybridization, the hotspot became accessible to an imager strand, yielding fluorescence enhancement factors of approximately 467-fold. Remarkably, the resulting signal could be visualized using a smartphone-based imaging setup, enabling portable single-molecule detection [69]. This strategy was subsequently applied to quantify antibiotic-resistance markers from *Klebsiella pneumoniae* at attomolar concentrations directly in human plasma, achieving polymerase chain reaction-level sensitivity without enzymatic amplification (Figure 4b). Beyond nucleic acid sensing, DNA–metal nanoantennas have been adapted for small-molecule detection by incorporating aptamer recognition elements. For example, ATP-binding aptamers integrated into DNA origami-templated Au@Ag nanostructures undergo target-induced conformational switching to form G-quadruplex structures, producing strong fluorescence enhancement and enabling single-molecule ATP detection [72].

In addition to static sensing, plasmonic DNA nanoantennas have enabled real-time observation of biomolecular dynamics. By confining fluorophores within plasmonic hotspots, photon emission rates are significantly increased, allowing acquisition of single-molecule FRET trajectories with temporal resolution improved by approximately one order of magnitude. This capability has facilitated direct observation of rapid folding transitions in intrinsically disordered proteins and short DNA duplexes, providing experimental access to transient states previously accessible mainly through simulations (Figure 4c) [70]. Collectively, these studies underscore the versatility of DNA nanostructure-mediated fluorescence platforms for both ultrasensitive biosensing and dynamic molecular analysis.

### 5.3. SERS-Based Biosensing

SERS-based biosensing benefits substantially from DNA-guided assembly strategies, which enable rational design of plasmonic nanostructures with programmable geometries and well-defined nanogaps. The structural precision of DNA nanotechnology allows fine control over gap size, nanoparticle composition, and analyte positioning, resulting in ultrasensitive, reproducible, and multiplexed Raman-based detection.

For nucleic acid analysis, DNA nanostructure-assembled SERS platforms have incorporated enzymatic target-recycling strategies to overcome sensitivity limitations. Duplex-specific nuclease-assisted SERS probes and exosome-targeted DNA–Au@Ag conjugates have been applied for the detection of miRNA-10b in serum and exosomal samples, achieving attomolar to femtomolar detection limits with high sequence specificity [73]. DNA nanostructure engineering has further enabled multiplexed miRNA profiling, where tetrahedral DNA probes or asymmetric core–shell nanoparticles functionalized with distinct Raman reporters allow simultaneous detection of multiple miRNA targets within a single sample [74].

**Figure 5:**
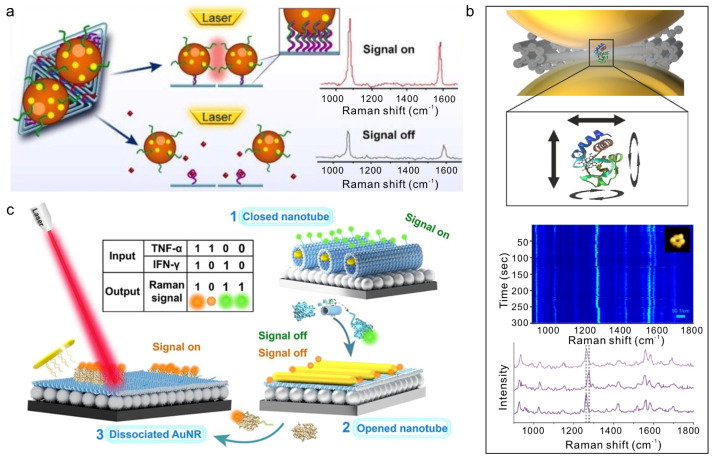
SERS-based biosensing (**a**) A DNA origami-assembled nanoantenna-based SERS biosensor for the ultrasensitive detection of trace amounts of diethylstilbestrol [75]. Copyright 2025, Royal Society of Chemistry. (**b**) A single-molecule SERS platform based on DNA origami nanoantennas for probing the dynamic conformational transitions of cytochrome c. [77]. Copyright 2024, American Chemical Society. (**c**) A programmable DNA origami plasmonic nanoantenna for multiplex SERS detection of TNF-α and IFN-γ relevant to cancer immunotherapy [78]. Copyright 2024, American Chemical Society.

Beyond nucleic acids, DNA origami provides a programmable scaffold for assembling plasmonic nanoantennas responsive to small molecules and proteins. Aptamer-functionalized origami templates decorated with AuNPs or AgNPs can transduce ligand binding into structural rearrangement, producing pronounced Raman signal modulation over wide concentration ranges (10^−10^–10^−5^ M) with sub-nanomolar detection limits (Figure 5a) [75,76]. Moreover, DNA-templated assembly of sharp-featured nanostructures, such as bimetallic Au@Ag nanostars, exploits strong tip-enhanced fields to enable sensitive detection of metabolites like pyocyanin at clinically relevant concentrations [59].

DNA origami-defined plasmonic hotspots have also enabled label-free single-protein identification and enzymatic analysis. By precisely positioning proteins such as thrombin or epidermal growth factor receptor within nanogaps, distinct single-molecule Raman fingerprints can be acquired [79]. Furthermore, real-time monitoring of enzymatic reactions has been achieved by immobilizing cytochrome C or horseradish peroxidase within DNA-defined hotspots, allowing catalytic processes to be tracked through Raman spectral evolution (Figure 5b) [77,80].

The programmability of DNA origami additionally facilitates multiplexed and logic-based biosensing. Stimuli-responsive nanoantennas incorporating Au nanorods and aptamer-functionalized origami sheets have been designed to detect cytokines such as TNF-α and IFN-γ, with Raman signal variations serving as Boolean logic outputs for immune-response profiling (Figure 5c) [78]. Integration of multiple optical modalities further enhances analytical robustness. For example, Y-DNA-mediated assembly of chiral AuNP core–satellite structures generate correlated SERS and CD signals, enabling dual-mode miRNA quantification in living cells [81]. Such multimodal platforms illustrate how DNA-assembled plasmonic nanostructures combine structural programmability, optical enhancement, and biochemical specificity to advance next-generation molecular diagnostics.

## 6. Conclusions and Future Perspectives

DNA nanostructure-guided assemblies of metal nanoparticles have emerged as a versatile and powerful platform for biosensing applications. The intrinsic programmability of DNA nanostructures allows precise spatial organization of nanoparticles, while dynamic scaffolds enable stimulus-responsive reconfiguration. Together, these features provide highly controlled, multifunctional, and tunable platforms capable of enhancing signal transduction, enabling multimodal detection, and implementing logic- or condition-dependent sensing.

Despite these remarkable advances, several challenges remain. The stability of DNA-nanoparticle assemblies under complex biological conditions, including nucleases, high ionic strength, or extreme pH, still requires further optimization. Large-scale, reproducible fabrication of uniform assemblies, particularly for hierarchical or multi-component architectures, remains technically demanding. In addition, integrating multiple signal transduction modalities within a single platform, while maintaining predictable and non-interfering outputs, presents both design and experimental challenges.

Looking forward, several opportunities are evident. Advancements in dynamic and responsive DNA nanostructures may enable more sophisticated control over nanoparticle arrangement, facilitating smart biosensing systems capable of autonomous signal processing and decision-making. Integration with microfluidic, wearable, or implantable devices could expand the practical applications of DNA nanostructure-guided nanoparticle biosensors in point-of-care diagnostics, environmental monitoring, and real-time cellular sensing. Moreover, combining DNA nanostructure-guided nanoparticle assemblies with other nanomaterials, such as quantum dots, upconversion nanoparticles, or 2D materials, may further enhance multimodal detection capabilities and functional versatility.

In summary, DNA nanostructure-assembled metal nanoparticles provide a unique combination of structural programmability, dynamic reconfigurability, and multifunctional signal transduction, offering a robust foundation for the next generation of biosensing technologies. Continued advances in scaffold design, assembly strategies, and integration with diverse signal modalities are expected to drive the development of highly sensitive, selective, and programmable biosensors for biomedical and environmental applications.

## Data Availability

No new data were created or analyzed in this study. Data sharing is not applicable.
